# bFGF Regulates PI3-Kinase-Rac1-JNK Pathway and Promotes Fibroblast Migration in Wound Healing

**DOI:** 10.1371/journal.pone.0012228

**Published:** 2010-08-17

**Authors:** Shigeyuki Kanazawa, Toshihiro Fujiwara, Shinsuke Matsuzaki, Kenta Shingaki, Manabu Taniguchi, Shingo Miyata, Masaya Tohyama, Yasuo Sakai, Kenji Yano, Ko Hosokawa, Tateki Kubo

**Affiliations:** 1 Department of Plastic Surgery, Osaka University Graduate School of Medicine, Osaka, Japan; 2 Department of Anatomy and Neuroscience, Osaka University Graduate School of Medicine, Osaka, Japan; University of Birmingham, United Kingdom

## Abstract

Fibroblast proliferation and migration play important roles in wound healing. bFGF is known to promote both fibroblast proliferation and migration during the process of wound healing. However, the signal transduction of bFGF-induced fibroblast migration is still unclear, because bFGF can affect both proliferation and migration. Herein, we investigated the effect of bFGF on fibroblast migration regardless of its effect on fibroblast proliferation. We noticed involvement of the small GTPases of the Rho family, PI3-kinase, and JNK. bFGF activated RhoA, Rac1, PI3-kinase, and JNK in cultured fibroblasts. Inhibition of RhoA did not block bFGF-induced fibroblast migration, whereas inhibition of Rac1, PI3-kinase, or JNK blocked the fibroblast migration significantly. PI3-kinase-inhibited cells down-regulated the activities of Rac1 and JNK, and Rac1-inhibited cells down-regulated JNK activity, suggesting that PI3-kinase is upstream of Rac1 and that JNK is downstream of Rac1. Thus, we concluded that PI3-kinase, Rac1, and JNK were essential for bFGF-induced fibroblast migration, which is a novel pathway of bFGF-induced cell migration.

## Introduction

The process of wound healing involves coordinated efforts of several cell types including keratinocytes, fibroblasts, endothelial cells, macrophages, and platelets. The migration, infiltration, proliferation, and differentiation of these cells cause an inflammatory response, which is essential for the formation of new tissue and lead to wound closure [1]. In this process, especially, fibroblast proliferation and migration play important roles in the formation of granulation tissue and wound closure.

Cell migration is indispensable for wound repair. Cell migration can be divided into multi-step cyclic processes. The basic migratory cycle includes extension of a protrusion, formation of stable attachments near the leading edge of the protrusion, translocation of the cell body forward, release of adhesions and retraction at the cell rear [2]–[4]. These steps require remodeling of the actin cytoskeleton, and the small GTPases of the Rho family are key regulators of these cytoskeletal dynamics. At the leading edge of the migrating cells, Rac1 induces the formation of lamellipodial protrusions via activation of the Wave complex [5], which provides the driving force of cell movements [6]–[8]. Cdc42 is involved in establishing polarity [9], and inhibition of Cdc42 can disrupt the directionality of migration [10], suggesting that Cdc42 can also contribute to the cell movement. On the other hand, RhoA promotes the contraction of actin stress fibers to generate contractile forces [11], [12]. However, RhoA is activated not only at the rear of migrating cells, but also at the front, implying that RhoA cooperates with Rac1 and Cdc42 to induce membrane ruffles [13].

The process of wound healing is regulated by numerous growth factors, such as epidermal growth factor (EGF), transforming growth factor-β, vascular endothelial growth factor (VEGF), platelet-derived growth factor (PDGF), and basic fibroblast growth factor (bFGF). bFGF is a member of a large FGF family of structurally related proteins that bind heparin or heparan sulfate and modulate the growth, differentiation, migration, and survival of a wide variety of cell types [14]. FGF binds to the different isoforms encoded by the four receptor tyrosine kinases designated FGFR1-4, and also binds to heparin or heparan sulfate proteoglycans. FGF-stimulation leads to recruitment of multiple Grb2/Sos complexes resulting in activation of the Ras/MAPK signaling pathway [15], and the Ras/MAPK signaling pathway plays an important role in bFGF-induced cell proliferation.

Regarding the role of the fibroblasts in the process of wound healing, bFGF is known to promote both proliferation and migration. Schreier et al. investigated the relative role of bFGF in migration and proliferation of fibroblasts in an *in-vitro* model of wound healing [16]. It was necessary to exclude the effect of bFGF on proliferation in their study to analyze the effect of bFGF on migration alone.

In the present study, we investigated the effect of bFGF on fibroblast migration during wound healing regardless of its effects on fibroblast proliferation, and we noticed signal transduction involving the small GTPases of the Rho family.

## Results

### Mitomycin-C blocked fibroblast proliferation

**Figure 1:**
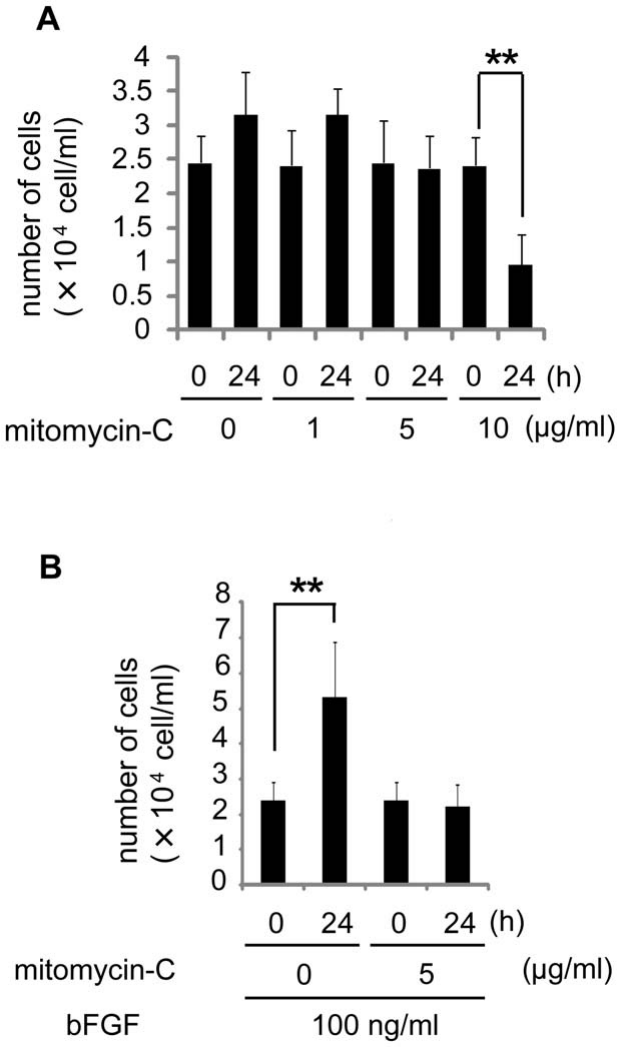
Number of the cells treated with each concentration of mitomycin-C. (A) We counted the number of fibroblasts in a microscopic counting chamber before or after treated with each concentration of mitomycin-C (0, 1, 5 or 10 µg/ml) for 24 h. Mitomycin-C at 0 or 1 µg/ml did not block cell proliferation. Mitomycin-C at 5 µg/ml completely blocked cell proliferation, while Mitomycin-C at 10 µg/ml was indicative of cellular cytotoxicity. (B) We counted the number of fibroblasts before or after treated with 0 or 5 µg/ml mitomycin-C in the presence of 100 ng/ml bFGF. Mitomycin-C at 5 µg/ml blocked bFGF-induced fibroblast proliferation. Data are mean ± s.e.m. of five independent experiments. **P<0.01, as compared with the control group (t test).

bFGF reportedly promotes fibroblast proliferation. Proliferation itself can promote wound healing [17], and therefore, it is necessary to exclude the effect on proliferation when evaluating bFGF-induced migratory ability in wound healing. Some investigators examined the effect of bFGF on fibroblast migration [18]–[21]. However, they did not exclude the effect on bFGF-induced fibroblast proliferation. Accordingly, we performed subsequent assays in the presence of mitomycin-C to block cell proliferation [16], [22], which enabled evaluation of cell migration with treatment of bFGF, while excluding any influence of cell proliferation. Firstly, to determine the optimal concentration of mitomycin-C to block cell proliferation, while not damaging the cell viability, we investigated the changes of fibroblast cell number 24 h after treatment with various concentrations of mitomycin-C. As shown in Fig. 1A, without bFGF, mitomycin-C at 1 µg/ml did not block cell proliferation. Mitomycin-C at 5 µg/ml completely inhibited cell proliferation, but did not decrease the cell number. Moreover, even in the presence of bFGF, mitomycin-C at 5 µg/ml blocked fibroblast proliferation (Fig. 1B). We therefore considered 5 µg/ml as the optimal concentration of mitomycin-C to block cell proliferation, and we performed all of the subsequent wound healing assays in the presence of mitomycin-C at 5 µg/ml.

### bFGF promoted fibroblast migration and induced lamellipodial extension

**Figure 2:**
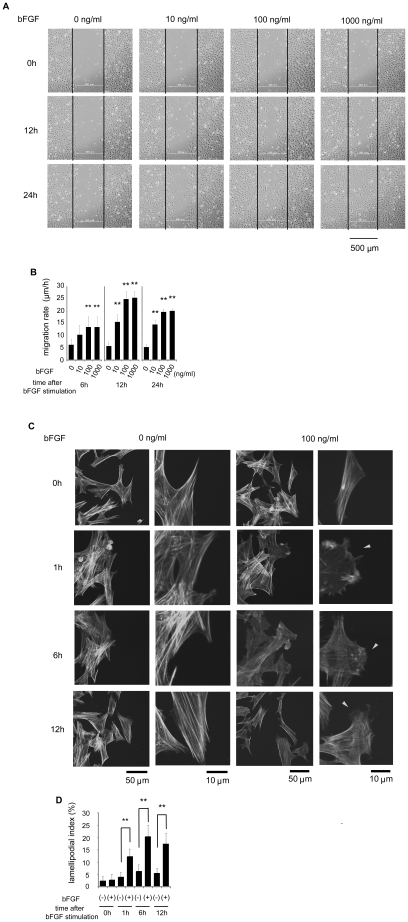
Effect of bFGF on fibroblast migration. (A) Wound healing assay in the fibroblasts treated with the indicated concentrations of bFGF in the presence of 5 µg/ml mitomycin-C. This photograph shows the wounded cell monolayers at 0, 12, and 24 h after wounding in the absence or presence of bFGF. The line indicates the wound edge at the start of the experiment (0 h). Bar  = 500 µm. (B) Analysis of the migration rate is expressed as migration distance/time (µm/h). (C) Immunocytochemistry of the cells at the margin of the scratch wound with rhodamine-conjugated phalloidin in the presence of 5 µg/ml mitomycin-C. This photograph shows the F-actin of the cell at 0, 1, 6, and 12 h after wounding with or without 100 ng/ml bFGF. The bFGF-treated fibroblasts developed lamellipodial extension (arrowhead). Bar  = 50 or 10 µm (D) Lamellipodial extension was quantified by summing the length of the outer margins of all lamellipodium in individual cells, and was expressed as a proportion of the total perimeter for each cell (lamellipodial index). Data are mean ± s.e.m. of five independent experiments. **P<0.01, as compared with the control group (t test).

To determine the optimal concentration of bFGF for fibroblast migration, we performed a wound healing assay for 24 h in the presence of mitomycin-C at 5 µg/ml with treatments by various concentrations of bFGF; namely, at 0, 10, 100, and 1000 ng/ml (Figs. 2A and B).

As shown in Fig. 2B, the cells treated with bFGF at 10 ng/ml showed an increased the migration rate during 12 h (15.63±1.04 µm/h) and 24 h (14.58±0.68 µm/h) as compared to the cells not treated with bFGF during 12 h (5.73±0.52 µm/h) and 24 h (5.34±0.36 µm/h) (see Movie S1 in the supporting information). The cells treated with bFGF at 100 ng/ml showed an increased the migration rate during 12 h (25.00±1.11 µm/h) and 24 h (19.79±0.39 µm/h) (see Movie S2 in the supporting information). The cells treated with bFGF at 1000 ng/ml showed an increased the migration rate during 12 h (25.52±0.94 µm/h) and 24 h (20.18±0.27 µm/h). These data indicated that bFGF at 100 or 1000 ng/ml had the greatest impact on promotion of fibroblast migration while not having any effect on fibroblast proliferation. We adopted 100 ng/ml of bFGF for the subsequent wound healing assays to promote fibroblast migration.

Migrating cells require remodeling of the actin cytoskeleton [2], [4], [23]. Therefore, we assessed the effect of bFGF on remodeling of the actin cytoskeleton by immunostaining the cells with rhodamine-conjugated phalloidin. As shown in Fig. 2C, the bFGF-treated fibroblasts significantly developed lamellipodial extension in the scratch wound. Lamellipodial extension was quantified by summing the length of the outer margins of all lamellipodium in individual cells, and was expressed as a proportion of the total perimeter of each cell (lamellipodial index), as described by Verma et al. [24]. bFGF induced lamellipodial extension at 1 h (12.17±1.30%), 6 h (20.33±1.91%), and 12 h (17.33±1.78%) after wounding as compared to the non-treated fibroblasts at 1 h (3.83±0.89%), 6 h (6.17±1.21%) and 12 h (5.50±0.85%) (Fig. 2D).

### bFGF activated RhoA and Rac1, but not Cdc42

**Figure 3:**
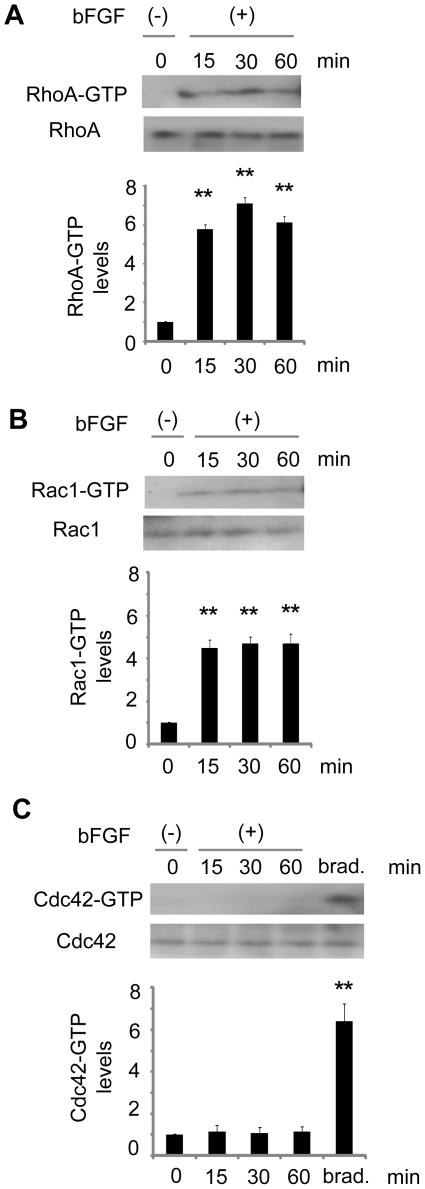
Activities of RhoA, Rac1, and Cdc42 by bFGF-stimulation. (A)(B)(C) Activities in pull-down assays for RhoA, Rac1, and Cdc42 were analyzed at 15, 30, and 60 min after 100 ng/ml bFGF-stimulation in the presence of 5 µg/ml mitomycin-C. Densitometry for RhoA, Rac1, and Cdc42-GTP was normalized to the amount of total RhoA, Rac1, and Cdc42. Bradykinin treatment (100 ng/ml) for 10 min was performed as a positive control of Cdc42 activation. The results are presented as fold change as compared with fibroblasts in the absence of bFGF. Data are mean ± s.e.m. of three independent experiments. **P<0.01, as compared with the control group (t test). brad. =  bradykinin.

The small GTPases of the Rho family, namely, RhoA, Rac1, and Cdc42, are the key regulators of cytoskeletal dynamics and cell migration. To investigate the involvement of RhoA, Rac1, and Cdc42 in bFGF-induced fibroblast migration, we measured the activities of RhoA, Rac1, and Cdc42 in the fibroblasts treated with bFGF (Figs. 3A, B, and C). As shown in Fig. 3A, RhoA activity increased 5.7-fold at 15 min after bFGF stimulation, with peak activation at 30 min and remained high for 60 min. Rac1 activity increased 4.5-fold at 15 min after bFGF stimulation and remained high for 60 min (Fig. 3B). However, Cdc42 activity did not increase with bFGF treatment (Fig. 3C). These data show that bFGF activated RhoA, Rac1, but not Cdc42.

### Rac1, but not RhoA, was essential for bFGF-induced fibroblast migration

**Figure 4:**
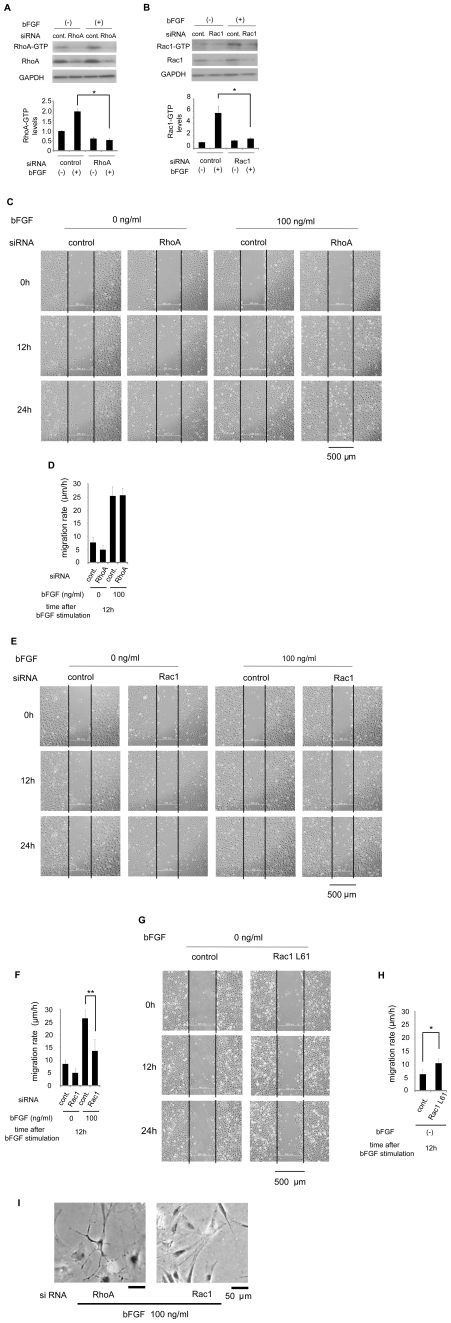
Rac1, but not RhoA, was essential for bFGF-induced fibroblast migration. (A)(B) Primary rat fibroblasts were transfected with 10 nM RhoA, 10 nM Rac1 siRNA or 10 nM nonspecific control pool siRNA. Pull-down assays for RhoA or Rac1 in the presence of 5 µg/ml mitomycin-C were performed after 48 h of siRNA treatment, using an immunoblot of lysates for GAPDH to verify equal protein loading. (C)(E) Wound healing assay containing 5 µg/ml mitomycin-C in fibroblasts transfected with 10 nM RhoA, 10 nM Rac1 siRNA or 10 nM nonspecific control pool siRNA in the presence or absence of bFGF. Bar = 500 µm. (D)(F)(H) Analysis of the migration rate of the RhoA, Rac1-inhibited cells, or the active Rac1-taransfected cells was performed. (G) Wound healing assay treated with 5 µg/ml mitomycin-C in fibroblasts transfected with 0.15 µg/cm^2^ of plasmid DNA (Rac1-61L) or empty vector as a control in the absence of bFGF. (I) These images show the cell shape of the RhoA or Rac1-inhibited cells at 24 h after wounding in the presence of 100 ng/ml bFGF. Bar = 50 µm. Data are mean ± s.e.m. of five independent experiments. *P<0.05, **P<0.01, as compared with the control group (t test).

Next, we examined how the inhibition of RhoA or Rac1 affects fibroblast migration (Figs. 4C and E). To block RhoA or Rac1, we used RNA interference with siRNA. As shown in Figs. 4A and B, transfection of RhoA siRNA or Rac1 siRNA led to a reduction in RhoA or Rac1 protein levels and did not show any activation in response to bFGF stimulation.

The migration rate of RhoA-inhibited cells was similar to that of the control cells during 12 h, regardless of the presence of bFGF (Figs. 4C and D). In contrast, Rac1-inhibited cells showed a reduction in the migration rate during 121 h (13.69±1.75 µm/h) as compared to the control cells in the presence of bFGF at 100 ng/ml (26.48±1.34 µm/h) (Figs. 4E and F). Thus, inhibition of RhoA did not affect bFGF-induced cell migration, whereas inhibition of Rac1 blocked bFGF-induced cell migration significantly. Furthermore, we investigated the effect of active Rac1 mutant on fibroblast migration. Figures 4G and H show that the active Rac1-transfected cells had increased the migration rate during 12 h (10.41±0.79 µm/h) as compared to the control cells in the absence of bFGF (6.25±0.85 µm/h). These data suggest that Rac1, but not RhoA, is essential for bFGF-induced fibroblast migration.

The small GTPases of the Rho family can also affect cell shape [6]. The cell shape of RhoA-inhibited cells changed to become round with prominent dendritic processes (Fig. 4I). On the other hand, the cell shape of Rac1-inhibited cells at the wound edge showed less formation of lamellipodium (Fig. 4I).

### Activation of PI3-kinase was involved in bFGF-induced fibroblast migration

**Figure 5:**
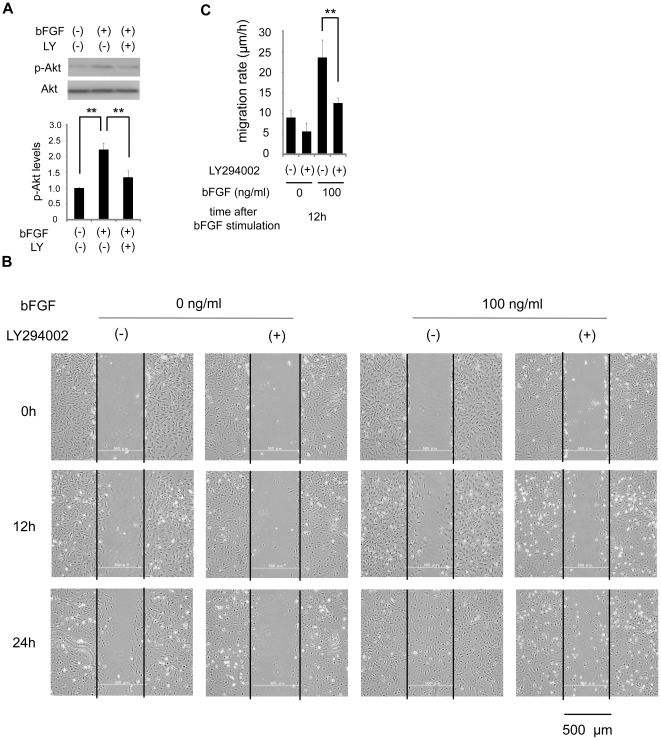
Effect of inhibition of PI3-kinase on fibroblast migration. (A) The activity of Akt with or without 10 µM LY294002 was analyzed by immunoblotting at 30 min after 100 ng/ml bFGF-stimulation in the presence of 5 µg/ml mitomycin-C. Densitometry for p-Akt was normalized to the amount of Akt. The results are presented as fold change as compared with the fibroblasts in the absence of bFGF. (B) Wound healing assay containing 5 µg/ml mitomycin-C in fibroblasts treated with or without 10 µM LY294002 in the presence or absence of 100 ng/ml bFGF. Bar  = 500 µm. (C) Analysis of the migration rate of PI3-kinase-inhibited cells was performed. Data are mean ± s.e.m. of five independent experiments. **P<0.01, as compared with the control group (t test).

Chemotactic migration is regulated by phosphoinositide 3-kinase (PI3-kinase) [25]–[28]. Therefore, we investigated the involvement of PI3-kinase in bFGF-induced fibroblast migration. Firstly, we measured the activation of the PI3-kinase effector Akt/protein kinase B in response to bFGF stimulation (Fig. 5A). Secondly, we performed a wound healing assay with LY294002, a PI3-kinase inhibitor, to examine how the inhibition of PI3-kinase affects fibroblast migration (Figs. 5B and C).

The relative level of the phosphorylated Akt increased at 30 min after bFGF stimulation (Fig. 5A). These data indicate that bFGF activated PI3-kinase and phosphorylated Akt. In the wound healing assay (Figs. 5B and C), PI3-kinase-inhibited cells in the presence of 100 ng/ml bFGF showed a reduction of migration during 12 h (12.50±0.54 µm/h) as compared to the control cells in the presence of bFGF at 100 ng/ml (23.61±1.76 µm/h). Thus, inhibition of PI3-kinase blocked bFGF-induced cell migration significantly. In addition, LY294002 inhibited bFGF-induced Akt phosphorylation (Fig. 5A).

### Activation of JNK is involved in bFGF-induced fibroblast migration

**Figure 6:**
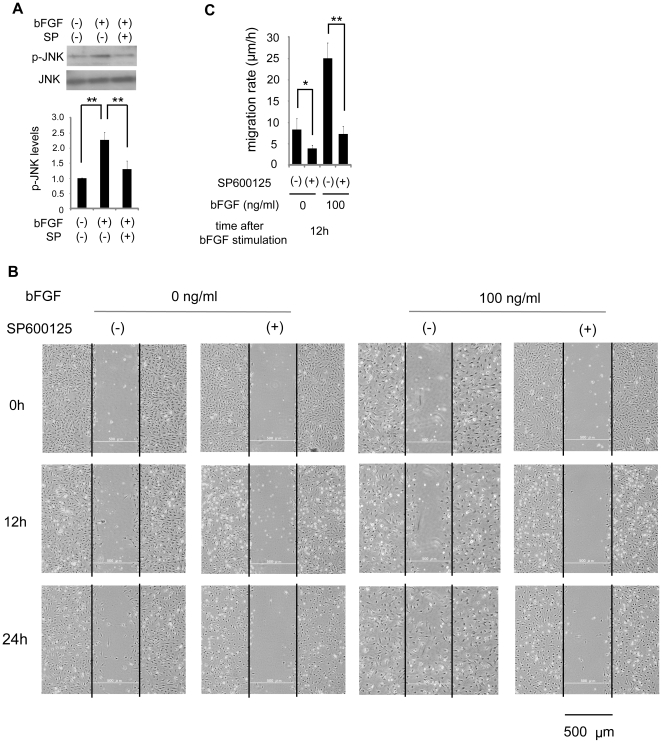
Effect of inhibition of JNK on fibroblast migration. (A) The activity of JNK with or without 10 µM SP600125 was analyzed by immunoblotting at 30 min after 100 ng/ml bFGF-stimulation in the presence of 5 µg/ml mitomycin-C. Densitometry for p-JNK was normalized to the amount of JNK. The results are presented as fold change as compared with fibroblasts in the absence of bFGF. (B) Wound healing assay containing 5 µg/ml mitomycin-C in fibroblasts treated with or without 10 µM SP600125 in the presence or absence of 100 ng/ml bFGF. Bar  = 500 µm. (C) Analysis of the migration rate of JNK-inhibited cells was performed. Data are mean ± s.e.m. of five independent experiments. *P<0.05, **P<0.01, as compared with the control group (t test).

Some recent studies have implicated the importance of the c-Jun N-terminal kinase (JNK) pathway in regulation of cell migration [29]–[32]. Therefore, we hypothesized that JNK is involved in bFGF-induced fibroblast migration. To test this hypothesis, firstly, we measured the phosphorylation of JNK in response to bFGF stimulation (Fig. 6A). Secondly, we performed a wound healing assay with SP600125, a JNK inhibitor (Figs. 6B and C).

As shown in Fig. 6A, the relative level of the phosphorylated JNK increased at 30 min after bFGF stimulation, suggesting that bFGF activated JNK. However, bFGF-induced JNK activation was suppressed by SP600125 (Fig. 6A). In the wound healing assay (Figs. 6B and C), JNK-inhibited cells showed a reduction in the migration rate during 12 h in the presence of bFGF at 100 ng/ml (7.29±0.71 µm/h) as compared to the control cells (25.01±1.52 µm/h). Thus, inhibition of JNK blocked the bFGF-induced cell migration significantly.

### PI3-kinase contributed to bFGF-induced Rac1 activation

**Figure 7:**
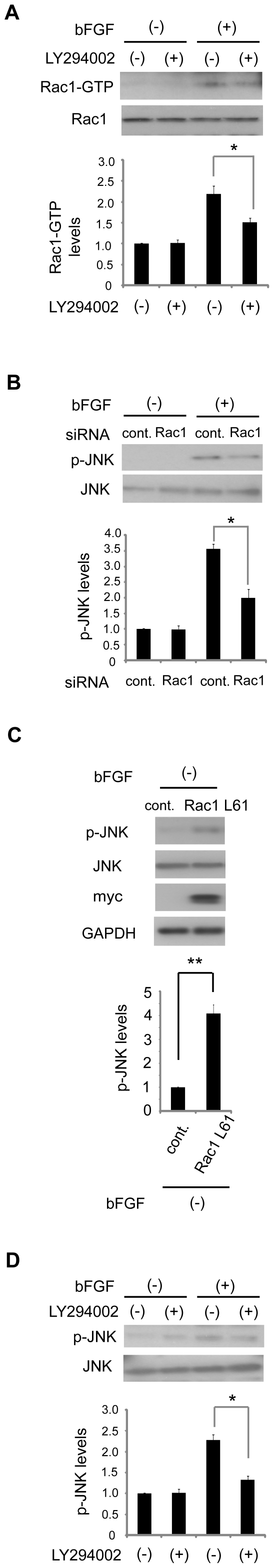
PI3-kinase contributed to bFGF-induced Rac1 and JNK activation. (A)(D) The activities of Rac1 or JNK in lysates of fibroblasts treated with or without 10 µM LY294002 were analyzed at 15 min after 100 ng/ml bFGF-stimulation in the presence of 5 µg/ml mitomycin-C. (B) The activity of JNK in lysates of fibroblasts transfected with 10 nM Rac1 siRNA or 10 nM nonspecific control pool siRNA was analyzed at 15 min after 100 ng/ml bFGF-stimulation in the presence of 5 µg/ml mitomycin-C. (C) The activity of JNK without bFGF in lysates of fibroblasts transfected with 0.15 µg/cm^2^ of plasmid DNA (Rac1-61L) or empty vector as a control was analyzed in the presence of 5 µg/ml mitomycin-C. Data are mean ± s.e.m. of three independent experiments. *P<0.05, **P<0.01, as compared with the control group (t test).

Rac1 reportedly acts as a downstream effector of PI3-kinase in several growth factor-stimulated pathways [33]. Accordingly, we measured the activation of Rac1 in fibroblasts treated with LY294002 in response to bFGF stimulation to examine whether PI3-kinase is upstream of Rac1 in bFGF-induced fibroblast migration. The level of Rac1-GTP showed a 45% reduction in the presence of LY294002 after bFGF stimulation (Fig. 7A), indicating that PI3-kinase contributed to bFGF-induced Rac1 activation.

### JNK was downstream of Rac1 in bFGF signaling

The activity of JNK is regulated by the small GTPases (Rac1 and Cdc42) [34], and Rac1 and JNK were also activated by bFGF in this study (Figs. 3B and 6A). Consequently, we measured the activation of JNK in the fibroblasts in the presence of Rac1 siRNA after bFGF stimulation to examine whether JNK is downstream of Rac1 in bFGF-induced migration signaling. As shown in Fig. 7B, the level of the phosphorylated JNK showed a 71% reduction in the presence of Rac1 siRNA. On the contrary, the active Rac1-induced cells showed a 4.1-fold increase of JNK phosphorylation as compared to the control cells (Fig. 7C). Finally, the cells treated with LY294002 also showed a 58% reduction in the phosphorylated JNK level (Fig. 7D). These data suggest that JNK was downstream of Rac1 and PI3-kinase in the signal transduction pathways of bFGF-induced fibroblast migration.

## Discussion

Migration of dermal fibroblasts is crucial for skin wound repair. Fibroblasts proliferate and migrate to the wound area, synthesize a new extracellular matrix, and contribute to wound healing [35]. Previous studies showed that bFGF induced cell migration in some cell types, such as the endothelial cells, keratinocytes, vascular smooth muscle cells and fibroblasts [16], [18]–[21], [36]–[39,]. However, the mechanism of bFGF-induced fibroblast migration is still unclear. Herein, we investigated the mechanism of the fibroblast migration by bFGF regardless of the effect on fibroblast proliferation, and we noticed the involvement of the small GTPases of the Rho family, PI3-kinase, and JNK.

Firstly, we demonstrated that the cells treated with bFGF at each concentration increased the migration speed significantly in the presence of mitomycin-C to block the cell proliferation (Figs. 2A and B). Moreover, immunocytochemistry showed that the bFGF-treated fibroblasts developed lamellipodial extension after wounding (Figs. 2C and D). This lamellipodial extension provides the driving force for the protrusive activity required for cell migration [6]. Therefore, our data suggest that bFGF induced lamellipodial extension of the fibroblasts and promoted migration.

Early changes in the actin cytoskeleton in the bFGF-mediated scratch wound repair model were characterized by Wang and Gotlieb in the endothelial cells [40]. They demonstrated that bFGF-treated cells at the leading edge were more elongated and had more prominent perpendicular actin microfilaments as compared to the non-treated cells at 4 h after wounding. In our study, the bFGF-treated fibroblasts showed more lamellipodial extension at 1 h after wounding as compared to the control cells. Those cells at 6 h after wounding showed more lamellipodial extension and increased migration speed (Figs. 2C and D). Consistent with Wang and Gotlieb's findings, we noticed that some of the cells treated by bFGF developed perpendicular actin microfilaments. The endothelial cells strongly bind with adjacent cells and migrate toward the wound as a cluster. However, the fibroblasts do not bind with other cells, and consequently migrate as individual cells (see Movie S2 in the supporting information). Therefore, the fibroblasts can move omni-directionally. This is presumably because the perpendicular actin and elongated cells were seen in only some bFGF-treated fibroblasts, not in all cells such as endothelial cells.

Secondly, we investigated which Rho GTPases, the key regulators of these cytoskeletal dynamics [10], [41], [42], were involved in bFGF-induced fibroblast migration. Our data demonstrated that bFGF activated RhoA, Rac1, but not Cdc42 (Figs. 3A, B, and C). Welsh et al. described the activation of Cdc42 by bFGF as well as integrins in hα5-3T3 cells. The differences between Cdc42 activation by bFGF in their study and in our study may be attributed to the differences of cell type and coating material of the culture dish. We used primary rat skin fibroblasts and plated them on a non-coated dish. However, Welsh et al. used hα5-3T3 cells, stably expressing an α5 human β1 mouse chimeric integrin, and plated the cells on a fibronectin-coated dish. Previous studies showed that integrins could activate not only their own downstream signals but also other growth factor receptors, such as the receptors of PDGF, EGF, VEGF and FGF [43]–[50]. Integrins and many growth factors can collaborate directly at the levels of tyrosine kinase receptor phosphorylation and their downstream signaling including small Rho GTPases [51]–[55]. Several reports also provide evidence for direct crosstalk between bFGF and integrin α5β1 [49], [50]. Therefore, bFGF-integrin cooperation may cause Cdc42 activation in hα5-3T3 cells on fibronectin-coated dishes.

Our data showed that inhibition of Rac1 blocked the bFGF-induced cell migration significantly (Figs. 4E and F). Accordingly, we concluded that Rac1 was involved in bFGF-induced fibroblast migration. The contribution of RhoA and Rho-kinase, a downstream regulator of RhoA, to cell migration depends on the cell types [56]–[60]. Several studies revealed that inhibition of RhoA/Rho-kinase in the neutrophils and monocytes inhibited cell migration [56], [57]. On the other hand, inhibition of RhoA/Rho-kinase promoted cell motility in other cell types; Swiss-3T3 cells, aortic smooth muscle cells and focal adhesion kinase-null cells [58]–[60]. Our study demonstrated that inhibition of RhoA did not affect bFGF-induced cell migration (Figs. 4C and D), while RhoA was also activated by bFGF-stimulation (Fig. 3A). This is presumably because of the balance between Rac1 and RhoA activation. Both Rac1 and RhoA were activated in the bFGF pathway, but activation of Rac1 may be dominant to RhoA activation. Indeed, a previous study revealed the importance of the balance between Rho GTPases in determining the morphology of neurons, as their balance affects the morphology of axons and dendrites, and spine growth [61].

PI3-kinase and JNK play important roles in the regulation of cytoskeleton dynamics and cell migration [28]–[32], [62]–[65]. Our study demonstrated that bFGF activated PI3-kinase and JNK (Figs. 5A and 6A), and that their inhibition blocked bFGF-induced cell migration significantly (Figs. 5C and 6C), suggesting that bFGF-induced fibroblast migration requires PI3-kinase and JNK activation.

**Figure 8:**
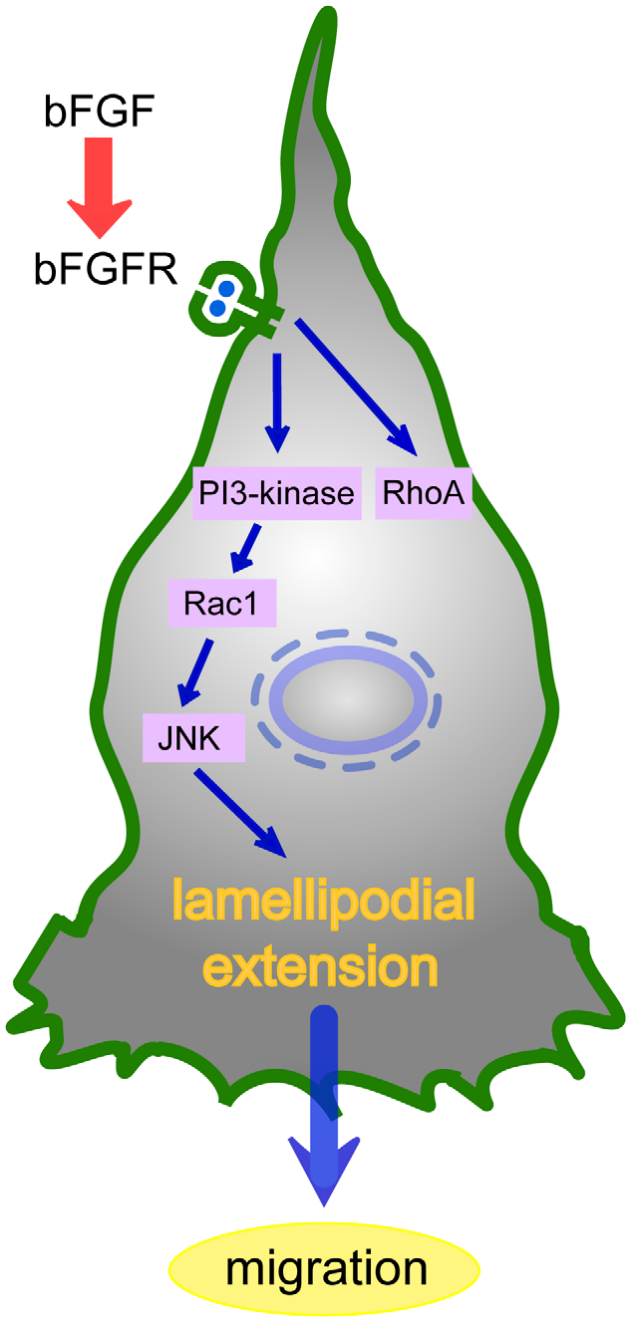
Signal transduction pathway of bFGF-stimulated fibroblast migration. Activated FGFR stimulates PI3-kinase. Activation of PI3-kinase leads to up-regulation of Rac1, followed by the activation of JNK, resulting in formation of lamellipodial extension.

Rac1 activation is often dependent on PI3-kinase activity, and inhibitors of PI3-kinase block Rac1 activation [66]–[69]. Moreover, previous studies suggested that the small GTPases Rac1 and Cdc42 regulate the activity of JNK signaling [26], [69]. Therefore, we assumed that bFGF promoted fibroblast migration through the PI3-kinase-Rac1-JNK pathway. Our data showed that inhibition of PI3-kinase with LY294002 down-regulated the activities of Rac1 and JNK via bFGF-stimulation (Figs. 7A and D), suggesting that PI3-kinase contributed to bFGF-induced Rac1 and JNK activation. Furthermore, inhibition of Rac1 by Rac1 siRNA down-regulated JNK activation (Fig. 7B), while the constitutively active Rac1-induction up-regulated JNK (Fig. 7C). These findings suggest that JNK is the downstream of Rac1. Finally, we concluded that bFGF promoted fibroblast migration through the PI3-kinase-Rac1-JNK pathway, which is a novel pathway of bFGF-induced cell migration (Fig. 8).

## Materials and Methods

### Cell culture

Primary skin fibroblasts were derived from rats aged 3 days. The cells were cultured in Dulbecco's Modified Eagle Medium (DMEM) containing 10% fetal bovine serum (FBS), 100 U/ml penicillin and 100 µg/ml streptomycin in a humidified incubator at 37°C with 5% CO_2_ atmosphere. For experiments, the cells were used between two and three passages. All animal experiments were carried out in accordance with a protocol approved by the Institutional Animal Care and Use Committee of Osaka University (permit numbers, 21-012-0).

### Cell proliferation assay

Second-passage fibroblasts were plated at a density of 2.43×10^4^ cells/ml on 35 mm dish and incubated for 24 h in DMEM containing 10% FBS. Then, the cells were treated with various concentrations of mitomycin-C (namely, 0, 1, 5, 10 µg/ml) (Sigma Aldrich Japan, Tokyo, Japan) and incubated for 24 h in DMEM containing 0.5% FBS in the absence or presence of 100 ng/ml bFGF (Kaken Pharmaceutical Co., Ltd., Tokyo, Japan). The cells were detached by trypsinization, and the cell number was counted using a microscopic counting chamber. Data are mean ± s.e.m. of five independent experiments. **P<0.01, as compared with the control group (t test).

### Wound healing assay

Confluent cells were cultured in DMEM containing 0.5% FBS treated with 5 µg/ml mitomycin-C for 24 h and then wounded with a linear scratch 500 µm by a sterile pipette tip. Images of the wounded cell monolayers were taken using a microscope (model IX70; Olympus, Tokyo, Japan) at 0, 12, and 24 h after wounding and recorded for 24 h using a microscope (model IX-70; Olympus) equipped with a CCD Camera (CoolSNAP HQ; Nippon Roper, Chiba, Japan) and controlled by MetaMorph software (Universal Imaging Co., Ltd., UK). All experiments were performed in the presence of 5 µg/ml of mitomycin-C to inhibit cell proliferation. To observe the effect of bFGF on fibroblast migration, the cells were treated with various concentrations of bFGF (i.e., 0, 10, 100, 1000 ng/ml) just before wounding. To observe the effects of RhoA or Rac1 inhibition, the cells were treated with siRNA before the wounding. To observe the effects of PI3-kinase or JNK inhibition, the cells were treated with 10 µM LY294002 (Jena Bioscience, Berlin, Germany) or 10 µM SP600125 (Calbiochem, San Diego, USA) for 60 min before the wounding. Twenty cells per one experiment (total 100 cells) at the wound area were randomly selected. At 12 h after wounding the distance between the selected 20 cells and wound edge at 0 h was measured by using the functions of “measurement length” in Image J software (National Institute of Health, Bethesda, MD, USA). Migration rate was expressed as migration distance/time (µm/h).

### Immunofluorescence

The fibroblasts were maintained in DMEM containing 10% FBS for 2 days and cultured in serum-free DMEM for 24 h. Then, the cells treated with or without 100 ng/ml bFGF for 0, 1, 6, and 12 h after wounding in the presence of 5 µg/ml mitomycin-C. The cells on coverslips were fixed with 4% paraformaldehyde in PBS for 15 min and washed with PBS five times. The cells were permeabilized with 0.3% Triton X-100 in PBS for 10 min and incubated with 5% bovine serum albumin (BSA) in PBS for 30 min to block nonspecific antibody binding. Then cells were incubated with the Alexa Fluor 594 phalloidin staining solution (5 U/ml, Invitrogen Japan, Tokyo, Japan) in PBS containing 1% BSA for 20 min and washed with PBS for 30 min. The coverslips were mounted onto the slides using VECTASHIELD Mounting Medium with DAPI (Vector Laboratories, Peterborough, England). Fluorescence images were taken using a confocal laser-scanning microscope (Axioplan2 LSM510; Carl Zeiss, Oberkochen, Germany). Lamellipodial extension was quantified by summing the length of the outer margins of all lamellipodium in individual cells. Each 20 cells per one experiment at the wound area were randomly selected. The perimeter of 100 ng/ml bFGF-treated cells or non-treated cells was measured by using the functions of “measurement perimeter” in Image J software (National Institute of Health, Bethesda, MD, USA). Lamellipodial index expressed as a proportion of the total perimeter for each cell.

### Western blot analysis and immunoprecipitation

The cells cultured in the presence of 5 µg/ml of mitomycin-C were lysed in RIPA buffer containing 1 mM Na3VO4, 1 mM NaF and Protease Inhibitor Cocktail (Roche Diagnostics, Basel, Switzerland), incubated for 20 min at 4°C and centrifuged at 15,000 *g* for 15 min at 4°C. The proteins were separated on SDS–PAGE and electrotransferred onto Immobilon-P Transfer Membranes (MILLIPORE JAPAN, Tokyo, Japan). The membranes were incubated in TBS containing 5% skim milk and 0.05% Tween-20 for 60 min and blotted with primary antibodies at 4°C overnight. An anti-phospho-Akt antibody (1∶1000, Cell Signaling Technology, Massachusetts, USA), anti-Akt antibody (1∶1000, Cell Signaling Technology), anti-phospho-JNK antibody (1∶1000, Cell Signaling Technology), anti-JNK antibody (1∶1000, Cell Signaling Technology), anti-GAPDH antibody (1∶2000, Abcam, Cambridge, USA) and anti c-Myc antibody (1∶100, Santa cruz biotechnology, California, USA) were used as primary antibodies. The membranes were incubated for 1 h with an anti-mouse or anti-rabbit HRP-linked secondary antibody (1∶2000, Cell Signaling Technology). Reaction products were visualized by detection of chemiluminescence using an ECL Western blotting Detection System (GE Healthcare, Piscataway, NJ, USA). Quantification of relative band densities was performed by scanning densitometry using Image J software (National Institute of Health, Bethesda, MD, USA).

### RhoA, Rac1, Cdc42 pull-down assay

Pull-down assays for Rho-GTPases were performed as described previously [66] with minor modifications. In brief, the cells were scraped into ice-cold lysis buffer containing 50 mM Tris-HCl, pH 7.4, 2 mM MgCl_2_, 1% NP-40, 10% glycerol, 100 mM NaCl, and Protease Inhibitor Cocktail (Roche Diagnostics, Basel, Switzerland) and centrifuged for 5 min at 14,000 *g*. The cleared lysates were incubated with 20 µg PAK-1 PBD agarose (Cytoskeleton, Denver, USA) or 20 µg GST-tagged Rhotekin Rho-binding domain bound to glutathione agarose (Cytoskeleton) for 60 min at 4°C. The beads were washed three times with lysis buffer and heated for 5 min at 100°C in reducing SDS-PAGE sample buffer, and then analyzed for bound RhoA, Rac1, and Cdc42 molecules by Western blotting using anti-RhoA antibody (1∶200, Santa cruz biotechnology), anti-Rac1 antibody (1∶500, Cytoskeleton), anti-Cdc42 antibody (1∶200, Santa cruz biotechnology). One hundred ng/ml bradykinin (Merck, Darmstadt, Germany), a Cdc42 activator, was used to show endogenously produced Cdc42-GTP.

### RNA interference experiments

siRNA duplex for the RhoA or Rac1 gene was synthesized by Invitrogen with help of the D-LUX Designer (Invitrogen Japan, Tokyo, Japan). The RhoA siRNA (5′-AUAAACCUCUGGGAACUGGUCCUUG-3′) was designed to target coding the region of rat RhoA mRNA sequence (GenBank accession no. NM_057132). The Rac1 siRNA (5′-CAGAAUAGUUGUCAAAGACGGUGGG-3′) was designed to target coding the region of rat Rac1 mRNA sequence (GenBank accession no. NM_134366). Primary rat fibroblasts were transfected with RhoA or Rac1 siRNA using Lipofectamine RNAiMAX (Invitrogen Japan) and Opti-MEM (Invitrogen Japan) by the manufacturer's protocol. The final concentration of siRNA was 10 nM. Stealth RNAi Negative Control Medium GC Duplex (Invitrogen Japan) was used as a control. The transfected cells were used for experiments after 48 h.

### Transient transfections

Myc-tagged Rac1-61L in pEF-BOS was a gift from Dr. T. Yamashita (Department of Molecular Neuroscience, Osaka University Graduate School of Medicine, Osaka, Japan). For transient transfection experiments, primary rat fibroblasts 60∼80% confluent were transfected with 0.15 µg/cm^2^ of plasmid DNA (Rac1-61L) or empty vector (pEF-BOS) using Lipofectamine2000 (Invitrogen Japan) and Opti-MEM (Invitrogen Japan) by the manufacturer's protocol. The transfected cells were used for experiments after 48 h.

### Supporting information

Images of the wounded cell monolayers were recorded for 24 h using a microscope (model IX-70; Olympus) equipped with a CCD Camera (CoolSNAP HQ; Nippon Roper) and controlled by MetaMorph software (Universal Imaging Co., Ltd.). All videos show time-lapse video recordings at 1 h intervals for 24 h. Movie S1 shows primary rat fibroblast migrating for 24 h in the absence of bFGF as a control. Movie S2 shows primary rat fibroblast migrating for 24 h in the presence of 100 ng/ml bFGF.

## Supporting Information

Movie S1 Fibroblast migration for 24 h in the absence of bFGF. Time-lapse image of the wounded fibroblast monolayers was recorded for 24 h in the absence of bFGF. Fibroblasts were treated with 5 µg/ml mitomycin-C before the experiment.
(5.06 MB AVI)

Movie S2 Fibroblast migration for 24 h in the presence of bFGF. Time-lapse image of the wounded fibroblast monolayers was recorded for 24 h after 100 ng/ml bFGF-stimulation. Fibroblasts were treated with 5 µg/ml mitomycin-C before the experiment.
(6.39 MB AVI)
